# Diagnostic imaging costs before and after digital tomosynthesis implementation in patient management after detection of suspected thoracic lesions on chest radiography

**DOI:** 10.1007/s13244-013-0305-1

**Published:** 2014-01-14

**Authors:** Emilio Quaia, Guido Grisi, Elisa Baratella, Roberto Cuttin, Gabriele Poillucci, Sara Kus, Maria Assunta Cova

**Affiliations:** 1 Department of Radiology, Cattinara Hospital, University of Trieste, Strada di Fiume 447, 34149 Trieste, Italy; 2 Department of Economics and Mathematical Sciences, University of Trieste, Piazzale Europa 1, 34127 Trieste, Italy

**Keywords:** Radiography, CT, Tomosynthesis, Lung, Chest, per-patient cost

## Abstract

**Objectives:**

To evaluate diagnostic imaging costs before and after DTS implementation in patients with suspected thoracic lesions on CXR.

**Methods:**

Four hundred sixty-five patients (263 male, 202 female; age, 72.47 ± 11.33 years) with suspected thoracic lesion(s) after CXR underwent DTS. Each patient underwent CT when a pulmonary non-calcified lesion was identified by DTS while CT was not performed when a benign pulmonary or extrapulmonary lesion or pseudolesion was identified. The average per-patient imaging cost was calculated by normalising the costs before and after DTS implementation.

**Results:**

In 229/465 patients who underwent DTS after suspicious CXR, DTS showed 193 pulmonary lesions and 36 pleural lesions, while in the remaining 236/465 patients, lesions were ruled out as pseudolesions of CXR. Chest CT examination was performed in 127/465 (27 %) patients while in the remaining 338/465 patients (73 %) CXR doubtful findings were resolved by DTS. The average per-patient costs of CXR, DTS and CT were €15.15, 41.55 and 113.66. DTS allowed an annual cost saving of €8,090.2 considering unenhanced CT and €19,298.12 considering contrast-enhanced CT. Considering a DTS reimbursement rate of € 62.7 the break even point corresponds to 479 DTS examinations.

**Conclusion:**

Per-patient diagnostic imaging costs decreased after DTS implementation in patients with suspected thoracic lesions.

**Main Messages:**

• *Digital tomosynthesis improves the diagnostic accuracy and confidence in chest radiography*

• *Digital tomosynthesis reduces the need for CT for a suspected pulmonary lesion*

• *Digital tomosynthesis requires a dose level equivalent to that of around two chest radiographies*

• *Digital tomosynthesis produces a significant per-patient saving in diagnostic imaging costs*

## Introduction

The detection and characterisation of pulmonary lesions, and particularly of pulmonary nodules, are challenging tasks on chest radiography (CXR) because of their frequently small size and poor conspicuity within surrounding anatomical structures. Pulmonary lesions are often visible only retrospectively when reviewing previous radiographic images of patients with known nodules [1], and computer-aided detection systems have been advocated to improve the diagnostic accuracy [2]. Frequently, the radiologist reporting CXR identifies doubtful or equivocal findings that could be referred to as both pulmonary and extra-pulmonary lesions or also as pulmonary pseudolesions because of different planes overlapping or composite areas of increased opacity.

Even though oblique radiographic views are still frequently employed, computed tomography (CT) is the gold standard for imaging pulmonary lesions, in particular pulmonary nodules [3, 4]. However, it is relatively expensive, delivers a considerable radiation dose to the patient and the diagnosis time can also be longer because of CT unit work overload. Therefore, CXR remains the initial examination for detecting most lung lesions, with CT being normally used to confirm the diagnosis in doubtful cases. As such, CT is frequently performed in patients without any pulmonary lesions or with pulmonary lesions that appear clearly benign or extra-pulmonary after CT [1, 3, 4].

Digital tomosynthesis (DTS) [5, 6] is a tomographic technique like CT, but delivers a lower radiation dose [5–8]. DTS is easily implemented in conjunction with CXR as it employs the same X-ray equipment. Unlike conventional tomography, DTS is not limited to reconstruction of a single plane, but can generate an arbitrary number of slice planes orthogonal to the projection direction. Reconstruction of the image planes is performed from a set of projection data acquired over a limited angle range of a single X-ray tube. A series of projection radiographs are acquired during the X-ray tube movement, and, due to the parallax effect, the anatomy at different depths in the patient can be mapped by the projections. These projections are then shifted and added to bring into focus objects in a given plane. By varying the amount of shift, planar images at different depths can be reconstructed [7–9], and objects outside of the focus plane are rendered with a varying amount of blur.

Previous studies have shown that DTS vs. CXR improved sensitivity in the detection of CT-proven lung nodules [7–10] and that DTS provides high diagnostic accuracy and confidence in confirming or ruling out pulmonary lesions suspected on CXR by improving pulmonary lesion conspicuity [11–14]. According to these results DTS could be considered a problem-solving technique in patients with suspected pulmonary lesions on CXR and could be used in the place of CT in this particular clinical setting. Cost analyses on different alternative diagnostic processes represent a valuable alternative for focussed cost reductions, instead of proportional fund reduction, and an opportunity for the measurement of the outcome of the diagnostic and therapeutic processes [15–18]. However, no previous studies have evaluated the actual impact in diagnostic imaging costs before and after DTS implementation.

The aim of this study was to evaluate diagnostic imaging costs before and after DTS implementation in patients with suspected thoracic lesions on CXR.

## Methods and materials

### Patients

This was a single-centre prospective study, approved by the ethics committee of our hospital, and informed consent was obtained from all patients after the nature of the procedure had been fully explained. From June 2007 to March 2012, all patients who revealed suspected pulmonary lesion(s) appearing as areas of increased opacity or pulmonary nodules [19] on CXR underwent DTS. CXR was obtained in the upright postero-anterior and left lateral views, and the preliminary CXR interpretation was performed on screen by two board-certified diagnostic radiologists affiliated to our department and respectively with 10 and 25 years of experience in thoracic imaging. Suspected pulmonary lesions were those that could not reliably be considered present or located within the lung, based only on CXR interpretation.

Eligibility criteria for the present study were: DTS performed within 15 days after CXR and absence of respiratory artefacts on DTS images preventing correct image assessment. Of the 506 patients who were deemed initially eligible for the study, 41 were excluded for the following reasons: (1) the patients had had previous chest surgery (*n* = 5); (2) the patients had subdiagnostic DTS images because of the inability to keep the upright position or suspend respiration (*n* = 7); (3) loss of patients’ information or incomplete patient follow-up (*n* = 29). Therefore, we finally included 465 patients (mean age 72.47 ± 11.33 years), who met the inclusion criteria including 263 men (mean age 71.48 years ± 10.58; median age 73 years; age range 23–94 years) and 202 women (mean age 73.74 years ± 12.13; median age 74 years; age range 34–97 years) and these patients were considered in the present study.

### Chest radiography

In each patient the CXR was obtained by a computed radiography (Kodak DirectView CR 975 System; Carestream, Rochester, NY, USA) or digital radiography (Definium 8000; GE Healthcare, Chalfont St Giles, UK). The X-ray tube had a focal spot size of 0.6 mm, a wall stand, and stationary anti-scatter grid (70 lines per cm; ratio 13:1).

### Digital tomosynthesis

The radiographical system (Definium 8000; GE Healthcare, Chalfont St Giles, UK) consisted of an X-ray tube (focal spot size 0.6 mm), a wall stand, a stationary antiscatter grid (70 lines per cm; ratio 13:1) and a cesium iodide–amorphous silicon (CsI/a-Si) indirect flat-panel detector (41 × 41 cm^2^; 200 × 200 μm^2^ pixel size). X-rays are converted to light in a layer of thallium-doped CsI, then the light is converted to electrical signals by a-Si photodiodes and the signal is multiplexed to the readout electronics by thin-film transistors (TFT) consisting of a-Si deposited on a glass substrate. The signal is digitised with 14-bit resolution (16,384 grey levels) by external electronics.

We employed the VolumeRAD option of the digital X-ray system, which acquires a series of very low-dose projection images during a single linear sweep of the X-ray tube over an angle of 30° with a stationary detector. A scout image is first obtained to check the patient position. If the scout image is satisfactory, the system calculates the appropriate low-dose exposure (mAs) for DTS. Technique factors were: voltage 120 kVp; detector entrance dose, 0.5 μGy; nominal focal spot, 0.6 mm; additional copper filtration, 0.1 mm; breath-hold acquisition time, 11 s; 60 low-dose projection images were acquired at regular angular intervals during the tube sweep. These data were then reconstructed with a filtered backprojection algorithm to generate a set of images at a 5-mm plane interval and a default number of slices depending on patient size (41, 53 or 61 slices for a body mass index <18.5, from 18.5 to 25 or >25, respectively). All projection images were used for the reconstruction of each plane.

### CT examination

CT of the chest was performed with a 64-row multi-detector CT (Aquilion, Toshiba, Tokyo, Japan). Patients were instructed to hold their breath with tidal inspiration during scanning. Technical parameters were: rotation time 400 ms; beam collimation 64 × 0.5 mm; normalised pitch 1; z-axis coverage 32 mm; reconstruction interval 0.3 mm; tube voltage 120 kVp; tube current (effective mAs) 180-250 mAs depending on patient size; field of view 40 cm^2^. Image reconstruction was performed in a 25–35-cm display field of view, depending on the patient’s body habitus. CT examinations consisted of an unenhanced scan followed by a vascular phase scan acquired 40 s after the intravenous bolus injection of iodinated contrast material (Iomeron 350, Bracco, Milan, Italy; 120 ml; 350 mg I/ml, 3 ml/s at 2 ml/kg followed by 50 ml of saline flush) administered with a dual-syringe power injector (Stellant CT injector, Medrad, Indianola, PA, USA) via a 20-gauge catheter inserted into an antecubital vein.

CT images at standard lung window settings (window level of -600 HU and window width of 2000 HU) were analysed immediately after image acquisition by a consensus of two senior radiologists with 8–15 years of experience in chest imaging affiliated to the radiology department where the study was performed but not involved in the visual interpretation of CXR or DTS images. Readers were free to scroll CT images and to employ coronal or sagittal reformations to measure the largest diameter of each thoracic lesion in each plane.

### Image analysis

Visual analysis of CXR and DTS images of each patient was carried out within 7 days after DTS by the same two radiologists who performed the initial CXR assessment. Image analysis was performed for patient care, and the CXR and DTS images of each patient were examined consecutively in the same reading session according to the routine diagnostic work flow. During the image analysis both readers worked independently and were aware of patient identification and clinical history, but were blinded to the other imaging findings. Readers were allowed to use processing tools such as windowing, image contrast, adjustment or magnification and to scroll the DTS images. All readings were performed on a picture-archiving and communications system (PACS)-integrated workstation by using a 19-inch thin-film transistor liquid crystal display (TFT LCD) with a resolution of 2,560 × 1,600 pixels at a central location with calibration of the luminance response. The first two cases, taken from the 465 cases included in the present study, were used for training to familiarise the radiologist with the process.

Readers were asked to re-identify those findings on the CXR images, which led each patient to undergo DTS. For those patients (*n* = 19) with more than one suspected pulmonary lesion on CXR, the largest and most conspicuous finding was considered as the marker lesion. Then, both readers were asked to confirm or exclude the same finding on DTS images and to express diagnostic confidence about lesion nature and pulmonary or extra-pulmonary location according to the scoring system reported on Table 1.

**Table 1:** Confidence scoring system

Confidence score	Reader finding
1 or 2	Definite or probable
	(1) Benign pulmonary^a^ or extrapulmonary lesion^b^
	(2) Pulmonary pseudolesion^c^
3	Indeterminate^d^
4 or 5	Probable or definite pulmonary lesion^e^

Diagnostic confidence scoring system

^a^Centrally calcified pulmonary lesions or pulmonary lesions with gross calcifications or calcified fibrotic scars with pulmonary architectural distortion

^b^Lesions not contained in the limits of lung parenchyma (e.g. pleura or thoracic wall) with or without calcifications

^c^Opacity not due to a true pulmonary or extra-pulmonary lesions but to normal anatomical structures including composite areas of increased opacity due to overlap of vascular and bone structures of the thoracic wall, vascular kinking, anatomic variant, or also to rib fracture, bone island or osteophytes

^d^Readers failed to classify confidently the presence of a lesion or whether a lesion was pulmonary or extra-pulmonary or was a pseudolesion

^e^A solid pulmonary lesion, a parenchymal or ground-glass opacity, or a solid or subsolid ground-glass pulmonary nodule

### Patient clinical management

The patient diagnostic workup was planned based on the readers’ confidence score for DTS images. Discrepant interpretations from the reader-independent analysis that could determine a potential change in the patient management were resolved by consensus through the involvement of an additional reader with similar experience in thoracic imaging. When DTS images were scored as 1 or 2, which is the identification of definite or probable benign pulmonary or extrapulmonary lesions or pulmonary pseudolesions, the imaging follow-up was perfomed by CXR, which was repeated after a mean time of 6 months (time range 3-8 months) from DTS. When DTS images were scored as 3, 4 or 5, which defined indeterminate or probable or definite pulmonary lesions, patients underwent CT within 1 week.

### Reference standards

When DTS images were scored as 1 or 2 (overt benign pulmonary or extrapulmonary lesions or pseudolesions), each patient underwent imaging follow-up consisting of a CXR planned a mean of 6 months (range 3-8 months) after DTS. Considering the imaging findings obtained by DTS, the imaging follow-up was stopped when CXR did not confirm any pulmonary lesion or confirmed the presence of overt benign pulmonary or extrapulmonary lesion.

When DTS images were scored as 3, 4 or 5, patients underwent contrast-enhanced CT within 1 week. All lesions presenting overt malignant features at CT (irregular or spiculated margins, pleural or vascular infiltrations) underwent surgical resection (*n* = 5 patients), while the remaining lesions were characterised by CT follow-up performed at least 6 months apart for a minimal period of 2 years. All lesions were considered benign if they contained fat or were calcified or subsequently disappeared during imaging follow-up, or decreased or remained unequivocally stable in size during serial examinations.

Those lesions appearing as ground-glass opacities on CT and that did not disappear during CT follow-up underwent CT-guided biopsies (*n* = 5 patients).

### Cost analysis

The observational time is from 1 January to 31 December 2012. We compared the unit full costs (€) of the different imaging units. All costs were derived from the accounting system of our hospital. All lead times and output measurements are expressed as the mean of the observed data [20]. According to the unit costs of the different imaging modalities we calculated the differential costs with and without DTS implementation based on patient number. We considered all the differential costs as the costs that would change if the hospital would implement an imaging modality in place of another imaging modality. In our case we compared an hospital with CXR and DTS to a second scenario in which only CXR and CT were available to image pulmonary lesions.

It is a reasonable assumption that the different imaging modalities do not determine any change in the common costs (such as administration, management and facility costs) and do not influence the final result [15, 21]. For this reason we considered only the long-term variable costs (personnel, depreciation, maintenance) besides the difference between the short-term variable costs [22]. We have included: capacity costs (personnel and fixed assets) and costs of non-capacity factors (variable costs) such as drugs, disposable devices, energy, prosthesis, etc. [15]. The utilisation cost of the fixed assets (depreciation), the medical, technical and nursing personnel costs are considered as capacity costs that are related to the specific activities through the mean lead times of use.

The personnel costs were calculated based on the medical, technical and nursing wage costs. We considered the mean wages based on the national contract, and the mean number of working days and hours to obtain the mean daily cost for each personnel category. The personnel costs were calculated based also on the unit occupation time, which was recorded for each patient and included the time for image reconstruction both for DTS and CT.

Every unit cost for each imaging technique (CXR, DTS, and CT) was computed on the basis of the lead times of capacity resources (e.g., personnel and equipments). Cases with subdiagnostic DTS images (7 patients) were included in the calculation of costs. We did not calculate any unit cost by dividing the fixed cost of capacity resources by the total number of imaging examinations for each technqiue. Better, we considered the support capacity management since every single examination does not require more time and costs whether the number of examinations decreases. There is no waste of capacity since other CT scans for other clinical reasons are actually performed in the place of those chest CT scans considered not necessary because of DTS.

The break even point for the DTS investment was calculated as:

- *Capacity (fixed) cost/unit contribution margin*
  where the capacity cost was the additional cost for the Volume RAD software while the unit contribution margin = reimbursement rate - variable unit cost.
  If the variable unit cost is zero as in the case of DTS, the breakeven point is equal to the:
- *Fixed cost/reimbursement rate*.
  The depreciation cost of the employed fixed assets (or technology costs) is calculated on a straight-line basis. The basic value for the depreciation corresponds to the historical cost of the equipment. The useful life of the radiological equipments for the economic analysis is considered 10 years, which include both their technological obsolescence and the clinical effectiveness [15]. The maximum practical capacity may be considered equal to that of the personnel by accepting that the fixed assets used by the hospital have an embedded waste due to the absence of availability of the personnel for the whole day.
  In our case we have considered 2,600 h of maximum practical capacity per year for the CXR and DTS units, and 3,030 h per year for CT. The maintenance costs were the current costs of 2012. We calculated the variable costs related to the quality and quantity of the materials consumed (contrast agents, disposable devices, etc.) and of the services (e.g., electric energy) for the use of radiological equipment employment. They were valued at direct cost. For CT we performed separate calculations for unenhanced CT and contrast-enhanced CT.

### Statistical analysis

Statistical analysis was performed with a computer software package (Analyse-it, version 1.63, Analyse-it-software, Leeds, UK). A per-patient analysis was performed with the marker lesion considered for the calculation of sensitivity, specificity, positive and negative predictive values, and overall diagnostic accuracy.

To assess the improvement in observers’ performance in correctly diagnosing pulmonary lesions, McNemar’s test [23] was employed for sensitivity, specificity, and positive and negative predictive values and the chi-square test with Yates correction [23] for accuracy. The improvement in diagnostic confidence was assessed by receiver-operating characteristic (ROC) curve analysis by plotting the sensitivity (true-positive fraction) against 1-specificity (false-positive fraction). The area under each ROC curve was calculated by using a non-parametric method [24], and the method proposed by Hanley and McNeil [25] was employed to compare the areas under each ROC curve.

The weighted *κ* statistic was calculated to assess intra- and inter-group observer agreement [26]. Agreement was graded as poor (κ value <0.20), fair (≥ 0.20 and < 0.40), moderate (≥ 0.40 and < 0.60), good (≥ 0.60 and < 0.80) and very good (≥ 0.8 up to 1). The reader interpretation time for CXR and DTS was compared by Wilcoxon’s signed rank test. For all statistical tests, a *P* value < 0.05 was considered to indicate a statistically significant difference.

## Results

**Fig. 1:**
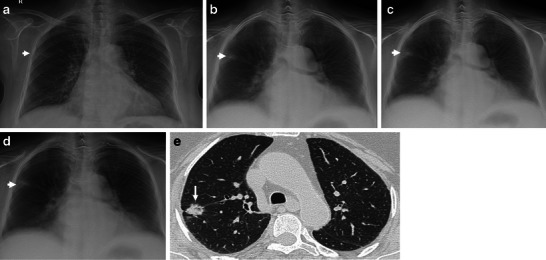
**a-e** A 60-year-old man with a suspected pulmonary lesion on the right lung. **a** Posteroanterior chest radiography in the upright position shows one suspected pulmonary nodule in the right lung (*arrow*). **b-d** Digital tomosynthesis images show the existence of a true lung opacity (*arrow*). **e** CT confirms the pulmonary opacity in the right lung (*arrow*)

Based on reference standards, a total number of 229 thoracic lesions, 193 pulmonary (Fig. 1) and 36 pleural, in 229/465 patients and 236 pseudolesions in the remaining 236/465 patients were included in the present study (Table 2).

**Table 2:** Imaging pattern of lesions

Diagnoses	*n*	Mean size (cm) ± SD	Size range (cm)
Pulmonary opacities	60	2.5 ± 0.3	2 - 3
Primary lung neoplasms^a^	5	2.5 ± 0.7	2 - 3
Ground-glass opacities/nodules^c^	47	2.5 ± 0.4	2 - 3
Non-calcified solid nodules^d^	32	1.1 ± 0.3	0.5 – 1.5
Pulmonary scars^b^	26	1.1 ± 0.3	0.5 - 1.5
Calcified solid nodules	23	1.1 ± 0.3	0.5 – 1.5
Pleural plaques	36	2.4 ± 0.6	1 - 3
Pulmonary pseudolesions ‡	236	-	-
Total number	465	2.3 ± 1.1	0.5 - 4

Radiographic patterns of lesions included in the present study according to reference standards

‡Includes composite areas of increased opacity resulting from overlap of vascular and bone structures of the chest (*n* = 129 patients), vascular kinkings (*n* = 55), prominent cardiac auricula (*n* = 32) or anatomical lung variants as accessory fissures (*n* = 20)

^a^Lung adenocarcinomas proven by histology and corresponding to a focal mass opacity with irregular or spiculated margins, and/or scissure or pleural or vessel infiltration on CT

^b^Areas of parenchymal bands with architectural distortion due to fibrosis with or without gross calcifications

^c^Ground-glass pattern as observed on CT, including two indolent lung adenocarcinomas proven by biopsy

^d^Includes two lung adenocarcinomas proven by histology

Table 3 reports the different values of diagnostic performance and confidence for CXR and DTS. CXR vs. DTS differed in sensitivity, specificity, positive and negative predictive values, and overall diagnostic accuracy and area under ROC curve for both readers. Inter-reader agreement improved from moderate with CXR (κ values = 0.40) to very good with DTS (κ value = 0.89). After resolution of discrepant interpretations between the two readers, chest CT examination was performed in 127/465 (27 %) patients while in the remaining 338/465 patients (73 %) CXR doubtful findings were resolved by DTS.

**Table 3:** Diagnostic performance and confidence

		CXR					DTS				
a											
Reader 1	Sensitivity (%)	24 (34/144)					80 (116/144)				
	Specificity (%)	10 (33/321)					95 (308/321)				
	Accuracy (%)	15 (69/465)					91 (424/465)				
	Diagnostic confidence:										
		Number	TP	TN	FP	FN	Number	TP	TN	FP	FN
	Score 1	1	/	1	/	/	295	/	295	/	/
	Score 2	35	/	32	/	3	15	/	13	/	2
	Score 3	379	/	/	303	73	17^a^	/	/	10	7
	Score 4	46	34	/	12	/	51	32	/	19	/
	Score 5	4	0	/	4	/	87	84	/	3	/
	(AUC) (95 % CI)	0.571 (0.525–0.616)					0.948 (0.924–0.967)				
b											
Reader 2	Sensitivity (%)	17 (25/144)					85 (122/144)				
	Specificity (%)	13 (43/321)					95 (308/321)				
	Accuracy (%)	17 (78/465)					92 (430/465)				
	Diagnostic confidence:										
		Number	TP	TN	FP	FN	Number	TP	TN	FP	
	FN										
	Score 1	2	/	1	/	1	297	/	296	/	
	Score 2	45	/	42	/	3	13	/	12	/	
	Score 3	386	/	/	304	82	17^a^	/	/	11	
	Score 4	31	25	/	6	/	31	22	/	9	
	Score 5	1	0	/	1	/	107	102	/	5	
	(AUC) (95 % CI)	0.612 (0.566–0.656)					0.947 (0.923–0.966)				

Visual prospective analysis in the pulmonary lesion diagnosis

The confidence scoring system is reported in Table 1

-*CXR* chest radiography; *DTS* digital tomosynthesis; *TP* true positive [lesion correctly assessed as a non-calcified pulmonary lesion (confidence score 4 or 5) or a lesion appearing as a parenchymal or ground-glass opacity, or a solid or subsolid ground-glass pulmonary nodule]; *TN* true negative (benign pulmonary lesion-centrally calcified lesion or lesion with gross calcifications or calcified fibrotic scars with pulmonary architectural distortion-or extra-pulmonary lesion or as a pulmonary pseudolesion; confidence levels 1, 2); *FP* false positive (benign pulmonary or extra-pulmonary lesion, or a pulmonary pseudolesion incorrectly assessed as a pulmonary lesion) (confidence levels 4 or 5) or assessed as indeterminate (confidence score 3); *FN* false negative (pulmonary lesion which should deserve further CT assessment assessed as an overt benign pulmonary lesion or pseudolesion or extrapulmonary lesion) (confidence levels 1 or 2) or assessed as indeterminate (confidence score 3); *NPV* negative predictive value; *PPV* positive predictive value. *AUC* area under the receiver-operating characteristic curve; *CI* confidence interval

Sensitivity was defined as TP/(TP + FN); specificity, as TN/(TN + FP): accuracy, as. (TP + TN)/(TP + TN + FP + FN)

All differences between chest radiography and digital tomosynthesis were statistically significant (*P* < 0.05)

^a^Including 7 (reader 1) or 6 (reader 2) solid subpleural pulmonary lesions and 10 (reader 1) or 11 (reader 2) pseudolesions

Based on DTS images, readers correctly classified all pseudolesions except for 10/236 (reader 1) or 11/236 (reader 2) pseudolesions and 7 (reader 1) or 6 (reader 2) pulmonary subpleural lesions (appearing as nodules or opacities both on CXR and DTS) in which they were not able to classify confidently whether a lesion was pulmonary or extra-pulmonary. Those subpleural lesions were located in the anterior or posterior region of the lung parenchyma in the proximity of the thoracic wall.

The mean interpretation time for DTS (103 ± 66 seconds; 47–290 seconds) was higher (*P* < 0.05) than for CXR (66 ± 23 seconds; 38–104 seconds).

Table 4 reports the equipment price, annual depreciation and maintenance costs.

**Table 4:** Full costs of the different equipments

	Equipment price	Annual equipment depreciation	Maintenance contract price
DTS	30,000^a^	3,750	10,000
CT	1,242,000	155,250	105,996

Costs are expressed in €

*DTS* digital tomosynthesis; *CT* computed tomography

^a^The cost includes the price for the VolumeRAD software, which should be added to the x-ray equipment including the flat panel detector and the x-ray tube

Table 5 reports the CXR, DTS and CT unit full costs. In the year before DTS implementation, 811 patients who presented suspected thoracic lesions underwent 271 CTs with an estimated annual cost of €17,709.85 for unenhanced CT and €30,801.86 for contrast-enhanced CT.

**Table 5:** Unit full costs (€) for the different imaging modalities

	Contrast agent	Medical personnel	Radiographers	Nursing	Asset depreciation	Total cost
CXR	0	11,65	3,05	0	0,45	15,15
DTS	0	23,04	7,92	0	10,59	41,55
Unenhanced CT	0	31,95	7,07	6,98	19,35	65,35
Contrast-enhanced CT	25,83^a^	46,24	11,19	11,05	19,35	113,66

Costs are expressed in €

The mean unit occupation time was 6 min (range, 4-9 min) for chest radiography, 13 min (range 9–16 min) for digital tomosynthesis, 15 min (range 11–16 min) for unenhanced computed tomography and 19 min (range, 15–22 min) for contrast-enhanced computed tomography

^a^Intended for 100 ml of contrast agent. €25.83 derives from the mean of €25.62 for iomeprol 350 and of €26.05 for iopromide 370

During the 12 months after DTS implementation, patients who presented suspected thoracic lesions underwent 91 CXR, 130 DTS and 39 CT with an estimated annual cost of €9,619.65 for unenhanced CT and 11,503.74 for contrast-enhanced CT considering also the 7 subdiagnostic DTS. Thus, the annual cost saving for the hospital with DTS is €8,090.2 considering unenhanced CT and €19,298.12 considering contrast-enhanced CT.

Considering that the reimbursement rate for DTS is €62.7 in our region and the variable costs are null for DTS, the breakeven point corresponds to 479 DTS examinations.

## Discussion

Numerous methods have been proposed to address perceptual limitations in CXR including dual-energy chest radiography, bone subtraction and computer-aided diagnosis (CAD), and these methods have experienced varying levels of success. One of the most recent and promising technological advancements for improving the diagnosis of subtle lesions in the chest is DTS [7–9, 18]. In comparison to CXR, DTS produces superior images for identifying the intra- or extrapulmonary lesions previously suspected based on initial CXR interpretation. The major advantages of DTS over conventional CXR are the removal of overlying anatomical structures, the enhancement of local tissue separation and availability of depth information of the structure of interest [5–7].

In this study DTS was confirmed to be decisive in confirming or ruling out pulmonary lesions and differentiating true pulmonary opacities from those due to pleural or thoracic wall lesions or pulmonary pseudo-lesions with a clear improvement in diagnostic accuracy, confidence and inter-reader agreement in comparison to CXR and with a modest increase in the radiation dose and interpretation time. This presents a strong clinical impact, since up to 20 % of suspected nodules on CXR can be entities mimicking a solitary nodule [27]. In our series, we found an even higher percentage of pulmonary pseudolesions (50 %) leading the patients to DTS. According to these results, DTS could be proposed as a problem-solving technique in the diagnosis of those suspected pulmonary lesions identified at the preliminary assessment of CXR.

In this study we showed that the per-patient diagnostic imaging costs decreased after DTS implementation in patients with suspected thoracic lesions. Mainly, this result was due to the dramatic effect of DTS on the CT utilisation rate that decreased significantly after DTS implementation. In fact, DTS resolved doubtful CXR findings for 338/465 (73 %) patients, reducing the need for CT to only 27 % (127/465) of patients. Once a chest opacity is suspected as being a solid pulmonary lesion by DTS, it should then go to CT for further evaluation, classification and staging. Lower CT utilisation translated into less radiation exposure for patients and to the availability of the CT unit for other examinations [28]. According to these results, DTS may be considered a first-line problem-solving imaging technique to rule out pulmonary lesions. Low-radiation-dose chest CT [28] could represent an alternative imaging modality, even though it appears more suitable for lung cancer screening [28–31], while the advantage of DTS is the verification of doubtful findings directly in the X-ray unit without moving the patient to CT and with a comparable effective dose to CXR and low-radiation-dose CT.

Compared to CT, the main limitation of DTS is the limited depth resolution caused by the limited tomographic sweep angle. Indeed, the resolution of DTS along the *z*-axis for chests may be approximated to 2 mm [32]. In our series, all findings misinterpreted at DTS were sub-pleural or located in the region of the lung in the proximity of the chest wall, where the limited depth resolution of DTS may hamper the correct spatial location of the findings. This limitation is related to the geometric frontal plane acquisition by DTS, since acquisition in the sagittal plane is not recommended because of the higher dose exposure [7, 8, 32].

DTS is a fast imaging technique that offers a clear improvement in diagnostic accuracy and confidence over CXR, with a much lower radiation dose than CT [28]. For the radiologist, DTS studies took longer to read than CXR mainly because of multiple image scrolling but the overall interpretation time was lower than CT because of the lower number of images evaluated. Even though DTS increased the interpretation time [28], it could be easily introduced in the routine diagnostic work flow as a case-solving technique in those patients with suspected or equivocal pulmonary lesions on CXR.

All costs were calculated based on a single hospital and are not necessarily transferable to other settings because of the difference in valued added tax (VAT) and personnel wages in our country. DTS could be interesting in de-centralised setting (e.g., northern European countries) where small sites would not have a CT scanner. In this case a digital x-ray unit could allow a further sparing by clarifying unclear pulmonary lesions without having the patient travelling far to the next centre with CT equipment. According to our findings, considering the fixed cost due to the Volume RAD software added to the digital detector and x-ray tube, the breakeven point for the DTS investment corresponds to 479 DTS examinations, which roughly corresponds to the number of patients included in the present series.

The principal limitation of our study was the inclusion of a heterogeneous group of patients who underwent CXR for different clinical reasons. The inclusion of a more selected patient cohort, e.g., patients with suspected pulmonary nodules or patients with a known primary tumour and suspected lung metastases, could provide further insights into the diagnostic accuracy and cost analysis of DTS. A second limitation is due to the absence of blinded evaluation of CXR and DTS images, since this prospective study was part of routine medical care. The third limitation is the presence of multiple reference standards including CXR for pseudolesions or overt benign lesions, and CT or histology for positive pulmonary lesions. This is because we decided to spare those patients who did not present any finding deserving further imaging workup on DTS from at CT dose and because this actually represents what usually happens in routine clinical practice.

In conclusion, DTS avoided the need for chest CT in about three-quarters of patients with suspected pulmonary lesions on CXR. Per-patient diagnostic imaging costs decreased after DTS implementation in patients with suspected thoracic lesions.

## Acronyms

CXR: chest radiography
DTS: digital tomosynthesis
CT: computed tomography
